# Optical limiting properties of surface functionalized nanodiamonds probed by the Z-scan method

**DOI:** 10.1038/s41598-018-36838-7

**Published:** 2019-01-24

**Authors:** O. Muller, V. Pichot, L. Merlat, D. Spitzer

**Affiliations:** 1 Laboratory for Radiation Interaction with Matter, French-German Research Insitute of Saint-Louis, 5 rue du Général Cassagnou, 68301 Saint-Louis, France; 2 NS3E “Nanomatériaux pour Systèmes Sous Sollicitations Extrêmes” UMR 3208 ISL/CNRS/UNISTRA, French-German Research Insitute of Saint-Louis, 5 rue du Général Cassagnou, 68301 Saint-Louis, France

## Abstract

This work focuses on the optical limiting behavior of surface modified nanodiamonds (DNDs) namely, amino-terminated DNDs (DND-NH2) and hydrogen-terminated DNDs (DND-H). Their relevant nonlinear optical properties for optical limiting are compared to those of unfunctionalized DNDs. The optical limitation is characterized by means of nonlinear transmittance, Z-scan, and scattered intensity assessments when submitted to a nanosecond pulsed Nd:YAG laser operating at a wavelength of 532 nm. It is stated that the largest nonlinear attenuation is attributed to the DND-H system, whereas the exceedingly low threshold values for optical limiting for the DND-H and the DND-NH2 systems is attributed to their negative electron affinity character (NEA). Using Z-scan experiments, it is shown that nonlinear refraction combined with a significant nonlinear absorption predominates in the DND-H suspension, while the pure thermal origin of the nonlinear refractive index change is conjectured in the case of the DNDs. Besides, an amazing valley to peak profile was measured on DND - NH2indicating an unexpected positive sign of the nonlinear refraction coefficient. In addition, a stronger backscattered intensity signal is highlighted for the unfunctionalized DNDs through nonlinear scattering measurements.

## Introduction

The application of nanodiamonds particles is widely spread out in biology^1–4^, pharmacology^5,6^, medicine^7,8^, or as a tuning agent for energetic compositions in pyrotechnics^9–11^. Besides, their nonlinear optical character has been proven since several studies relating their optical limiting properties have been reported^12–19^. Diamond, respectively nanodiamond is the densest allotropic form of carbon in which the atoms are networked through covalent sp^3^ bonds in a tetrahedral close packed crystalline lattice. Among the various ways to synthesize nanodiamonds, the shock wave or detonation method is one of the most versatile and cost effective with tunable properties and surface chemistry possibilities. In few words, detonation nanodiamonds (DNDs) are synthesized by detonation using a high explosive mixture composed of trinitrotoluene and hexogen. The detonation of the charge leads to a powder containing nanodiamonds as well as metallic impurities and sp^2^ carbon species. It follows a several step purification process leading to the end-user powder composed of nano-sized particles, typically 5 nm to 10 nm. A detailed study on the synthesis of DNDs and their purification method is given by Pichot *et al*.^20^. Generally speaking, DNDs offer a tenfold and tunable choice of surface functionalization strategies depending on the application and the desired physical and chemical properties of the surface. Analytical methods like interference-free reflectance-absorbance FTIR spectroscopy at Brewster’s angle^21^ or the Boehm tritration^22^ can be implemented to assess the functional groups on the DND surface. The most prominent surface functions are the hydroxyl (-OH), the carbonyl (>C=O), the amino (-NH2) and the carboxyl group (-COOH).

In this work, we aim to investigate the nonlinear mechanisms responsible for the optical limiting behavior of amino-terminated DNDs (DND-NH2), hydrogen-terminated DNDs (DND-H) and compare them to unfunctionalized DNDs. To our knowledge, the use of such chemically engineered DNDs for optical limiting applications has never been reported elsewhere. The laser source used in this study is a frequency doubled Nd-YAG emitting nanosecond duration pulses at a wavelength of 532 nm. In a recently published paper, we reported on the nonlinear optical properties of porphyrin-functionalized DNDs^15^. Albeit some works dealing with amino or hydrogen-terminated DNDs are related in the literature (see for example^23^, see also^24^ or^25^, respectively), there exists no study referring their nonlinear properties for optical limiting applications.

In Section 2 we present the nanomaterials, while the experimental details on the nonlinear optical measurements are given in Section 3 followed by the results statement and discussion in Section 4. Finally, Section 5 gives the conclusion of this work.

## Materials and Samples Preparation

**Figure 1:**
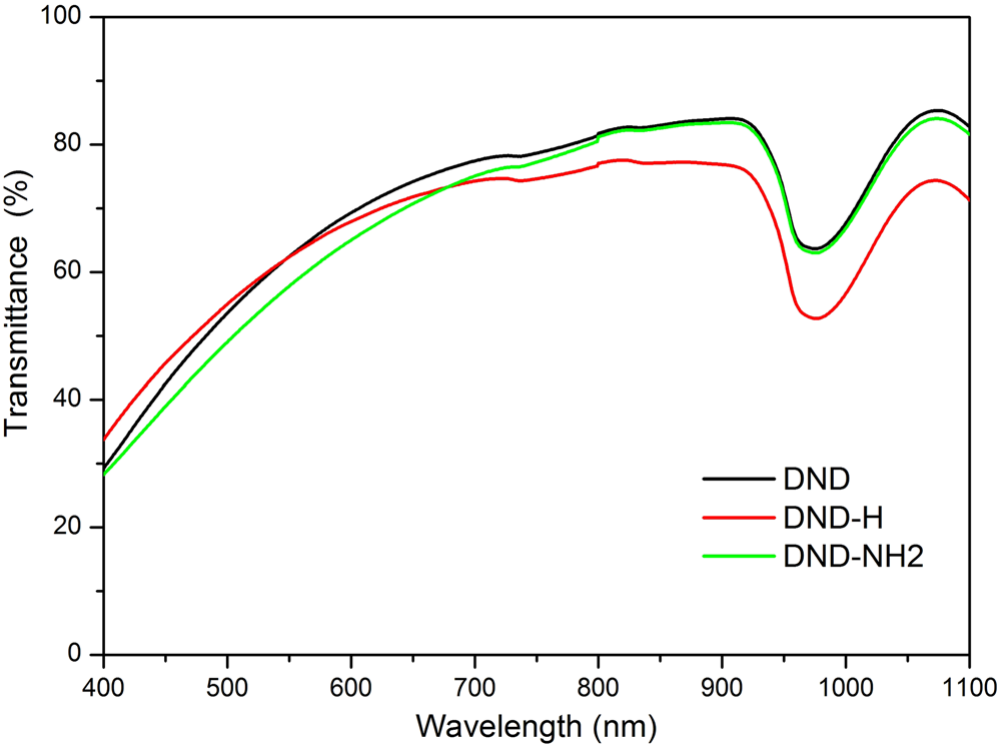
Linear transmittance spectra of the DND, DND-H and DND-NH2 suspensions in water.

The DNDs surface modification or functionalization was achieved by means of the native hydroxyl following the procedure detailed in a previous paper^14^. The DND samples were prepared as suspensions in ultrapure water (resistivity 15 MΩ.cm^−1^). During the course of the experiments, they were maintained in 10 mm path length quartz cuvettes except for the Z-scan measurements where we used 1 mm cuvettes. The solutions were prepared following the procedure described in^17^. Shortly, 1 g.l^−1^ nanodiamonds suspensions in water were organized and submitted to a 1 h ultrasonication. The supernatant (0.11 g.l^−1^) obtained from the latter suspensions after 24 hours sedimentation was subsequently ultracentrifugated at a relative centrifugal force of 8 × 10^3^ g. The linear transmittances of the resulting suspensions are shown on Fig. 1. At a wavelength of 532 nm we measured linear transmittances of 60% for the DND, DND-H and 55% for the DND-NH2. To be noticed that the strong absorption around λ = 980 nm is due to water.

The hydrodynamic size of the nanoparticles present in the supernatant was measured by dynamic light scattering (DLS) using a Malvern Zetasizer NanoZS. It is worth noting that we assume the particles as spheres, so that their real radius can be relatively well approximated with their equivalent hydrodynamic radius value. All of the nanoparticles present a radius of 60–70 nm as it can be seen in Table 1.

**Table 1:** Particle sizes in water as measured by DLS.

Material	Radius (nm)
DND	67
DND-H	66
DND-NH2	63

## Experimental Details

**Figure 2:**
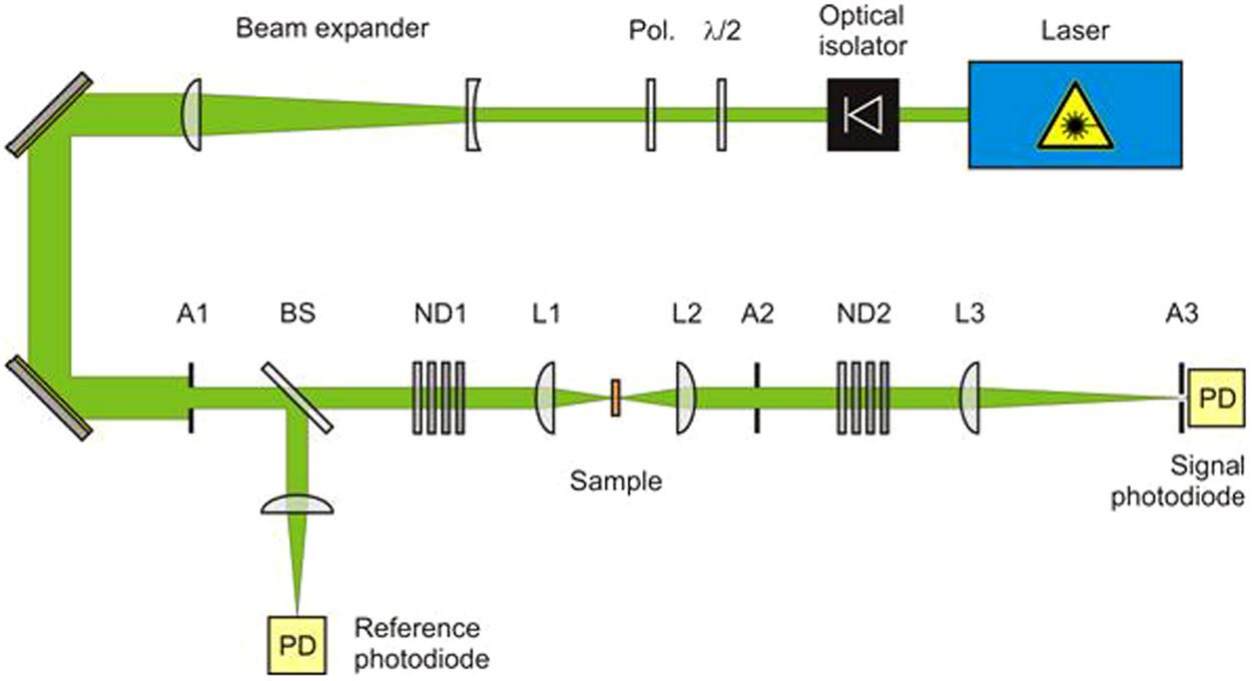
Experimental setup used to study the optical limiting behavior. A1 and A2, apertures (12 mm and 20 mm, respectively); BS, beam splitter;ND1 and ND2, neutral density filters; L1, L2 and L3, plano-convex lenses (focal lengths 60 mm, 100 mm and 400 mm, respectively); PD, photodiode.

For this work, we used a frequency-doubled, Q-switched Nd-YAG laser (Quantel) emitting at a wavelength of 532 nm with an output energy extending up to 160 mJ, a repetition rate fixed to 1 Hz and a pulse width of τ_p_ = 4 ns. The experimental setup used to assess the nonlinear transmittance is sketched in Fig. 2. To simulate far–field propagating waves conditions, the original laser beam was expanded with a 6.7 X Galilean telescope before entering the Keplerian one. The entrance aperture A_1_ was overfilled by the expanded beam so that a top-hat spatial irradiance distributed beam resulted. The nanomaterials samples were placed at the intermediate focal plane of the Keplerian telescope made of plano-convex lens L_1_ and L_2_ with focal lengths of 60 mm and 100 mm, respectively. In front of the input lens L_1_ and behind the output lens L_2_, the apertures A_1_ and A_2_ (12 mm and 20 mm, respectively) were placed to achieve an optical system with a f-number of f_n_ = 5. With the help of a beam profiler (Cohu CCD camera), the focal diameter was estimated to be 4 µm at 532 nm. A part of the laser beam is split off by the beam splitter BS to monitor the incident energy, whereas the laser beam transmitted through the sample is further focused using the plano-convex lens L_3_ (focal length 400 mm) to measure the signal energy. Additionally, the aperture A_3_ with an opening diameter of 600 µm is positioned at the focal point of L_3_ toward the signal photodiode. This combination ensures that the focusable energy is detected within a solid angle of 1.5 mrad corresponding to the smallest critical angle of the human eye (following DIN EN 60825-1). If a radiation is collected from a field of view of less than this critical angle, the source may be considered as a point source.

**Figure 3:**
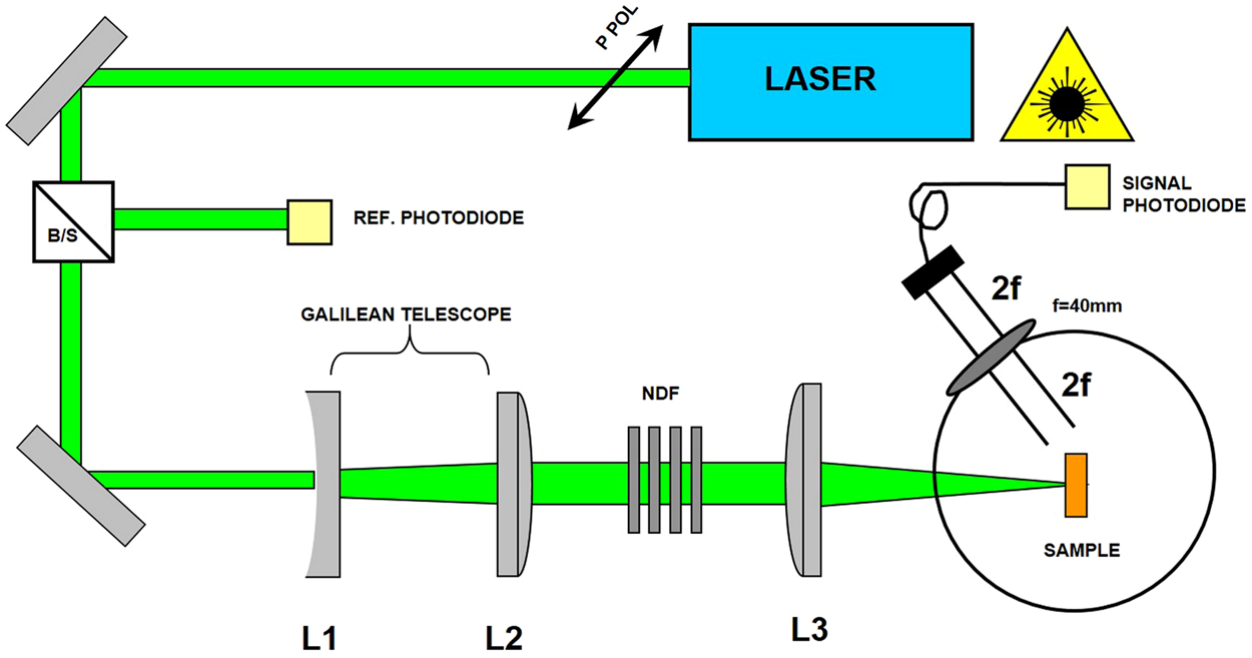
Experimental setup used to study the polar scattering properties. B/S, beam splitter; L_1_, negative focal lens, f_1_ = 60 mm; L_2_, positive focal lens, f_2_ = 375 mm; L_3_, plano-convex lens, f_3_ = 200 mm.

The setup used for the determination of the polar nonlinear scattering is sketched on Fig. 3. Experimental details on the setup are reported elsewhere^26^. Briefly, the beam, expanded by means of a Galilean telescope composed of a negative focal lens L_1_, f_1_ = 60 mm and a positive one L_2_, f_2_ = 375 mm, is focalized by the plano-convex lens L_3_, f_3_ = 200 mm. As a result, an optical system with a f-number f_n_ = 8 is achieved. The sample is located in the focal plane of L3 and the radius of the laser beam at the focus is 30 µm. The scattered signal is recorded on a photodiode (Thorlabs DET 36 AM). The detection unit was built on a rotating stage allowing the assessment of polar scattering diagrams from 20 dg to 155 dg; the sector [20 dg–90 dg] defines the forward scattering, whereas the sector [90 dg–155 dg] relates to the backscattering. Finally, the scattered signal from the sample is imaged at the entrance of an optical fiber via a 2 f optical system (f = 40 mm). The resulting input fluencies range from 1 J/cm² to ca. 70 J/cm².

**Figure 4:**
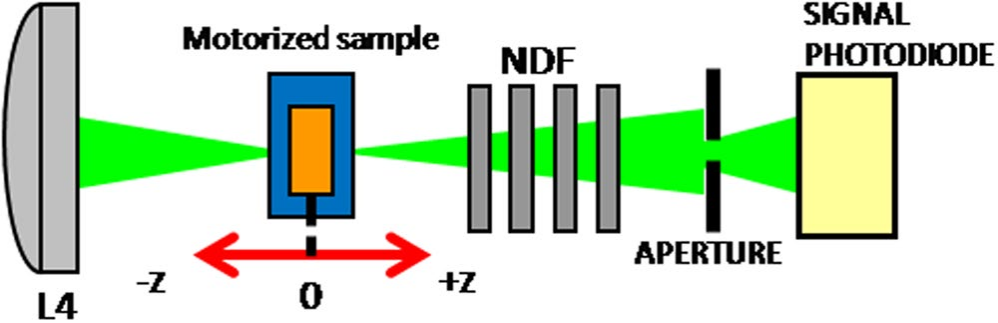
Z-scan optical setup. L4, plano-convex lens, f_4_ = 200 mm; NDF, neutral density filters; aperture hole, 600 µm.

A fraction of the incident beam is taken off from the main setup right before the Keplerian telescope (Fig. 2) and directed toward the Z-scan experimental setup sketched in Fig. 4.

In its open-aperture Z-scan configuration, an incident beam with a diameter of D_4_ = 7 mm is focused into the sample area through the plano-convex lens L_4_, f_4_ = 200 mm in a f_n_/30 focusing geometry^27–29^. The sample is mounted on a motorized stage and can be moved from −z, z = 0 (focal plane of L_4_) to +z. The emerging signal is collected on a DET 36 A/M photodiode (Thorlabs) before which we placed a 600 µm aperture to separate the nonlinear effects in a close-aperture Z-scan scheme. The use of 1 mm thin cuvettes follows the statement that the medium is to be considered as thin, i.e. a thickness smaller than the diffraction length of the focused beam^30^. Actually, if we assign L to the cell thickness and z_0_ to the diffraction length, the condition z_0_ > L has to be accounted for. The characteristic diffraction length is given by z_0_ = πw_0_²/λ, where λ designates the wavelength and w_0_ the beam radius at the waist. This parameter depends on the beam quality factor M² (M² = 1.86 in our case) through the relationship w_0_ = 2 M²λf_4_/(πD_4_). Combining both relations we obtain a diffraction length z_0_ = 1.9 mm so that the thin approximation medium is valid. It is worth noting that all the optics of Figs 2, 3 and 4 were especially AR treated at the wavelength of 532 nm.

## Results And Discussion

**Figure 5:**
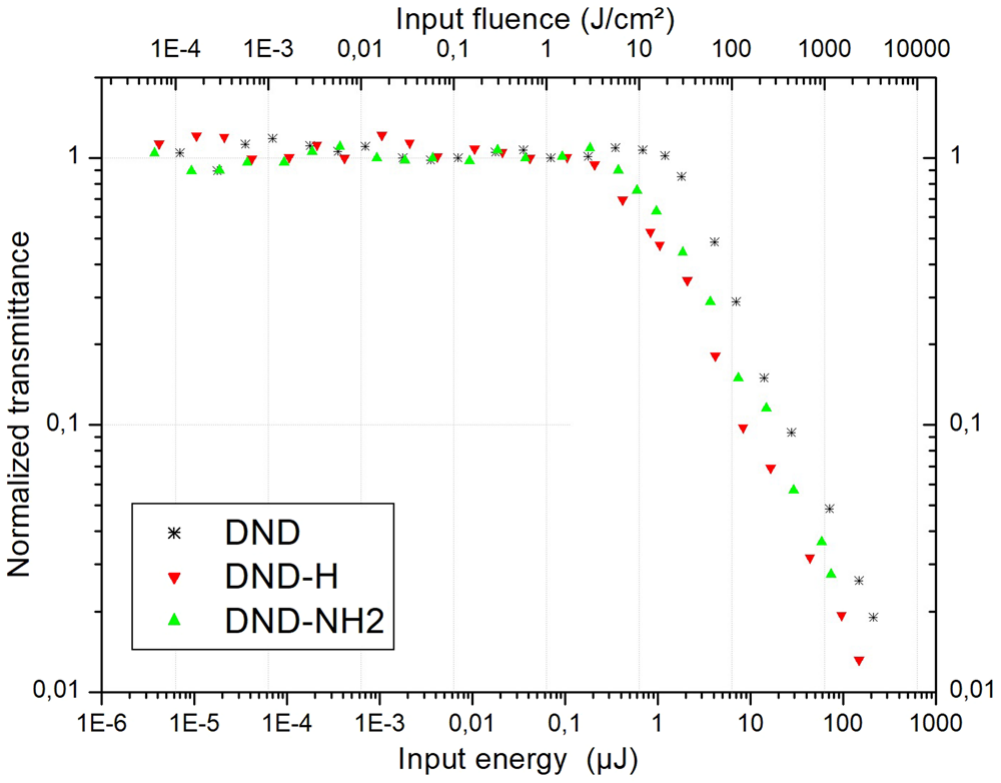
Normalized transmittance as a function of the input energy and input fluence in a log-log scale. DND, DND-H and DND-NH2 suspensions in water at λ = 532 nm are represented.

The normalized nonlinear transmittance results are presented on Fig. 5. The input fluence was defined to range from approximately 100 µJ/cm² to 3 × 10^3^ J/cm². The thresholds for optical limiting calculated in such a way where the transmittance drops to 50% are 65 J/cm² for the DND system, 15 J/cm² for the DND-H and 25 J/cm² for the DND-NH2 suspension. Considering the DND suspension, we notice that the calculated threshold is a factor 3 lower than the one obtained in a previous work^15^. It is very likely that different parameters such as the size of the aggregates, the polydispersity and the nature of the solvent used may explain this discrepancy. At the same time, it is very interesting to notice the extremely low threshold values for the DND-H and the DND-NH2 systems. It is rational to assume the inherence of such a behavior to the negative electron affinity (NEA) character of both materials^31,32^. This unusual aspect lies in the fact that the energy of the conduction band is above the vacuum level, therefore encouraging electron donation through appropriate surface dipoles and accordingly higher polarisability ability. In this way, it is probable that the emission of electrons from the DND-H or DND-NH2 systems into water could produce a reservoir of solvated electrons that could be responsible for the photoreduction of water. Albeit high-energy reduction reactions are most likely to be expected in the UV wavelength range as described by Zhu *et al*.^31^, the occurrence of multiphotons absorption processes at the wavelength of 532 nm (nanosecond regime) and the subsequent release of electrons in the medium is thoroughly credible. In an interesting work^33^, Petit *et al*. modeled the charge transfer from NEA-type DNDs in aqueous media and stated that electron accumulation at the diamond-water interface could happen. From the hydrophobic character of the H-terminated diamond, it results the formation of a hydrophobic gap at the diamond-water interface that enables charge accumulation^34^. We expect that accumulation of electrons and the subsequent strong polarization in DND-H and DND-NH2 systems contribute in the lowering of the thresholds for optical limiting. Regarding the optical limiting character, it is worth mentioning that none of the three systems under study presented a nonlinear attenuation greater than 2 orders of magnitude. Their level of nonlinear performance is lesser than the one reported for metallic nanoparticles^35^ or porphyrin-functionalized DNDs^15^, but comparable to the results published in studies on silver nanoprisms^36^ or graphene^37^.

**Figure 6:**
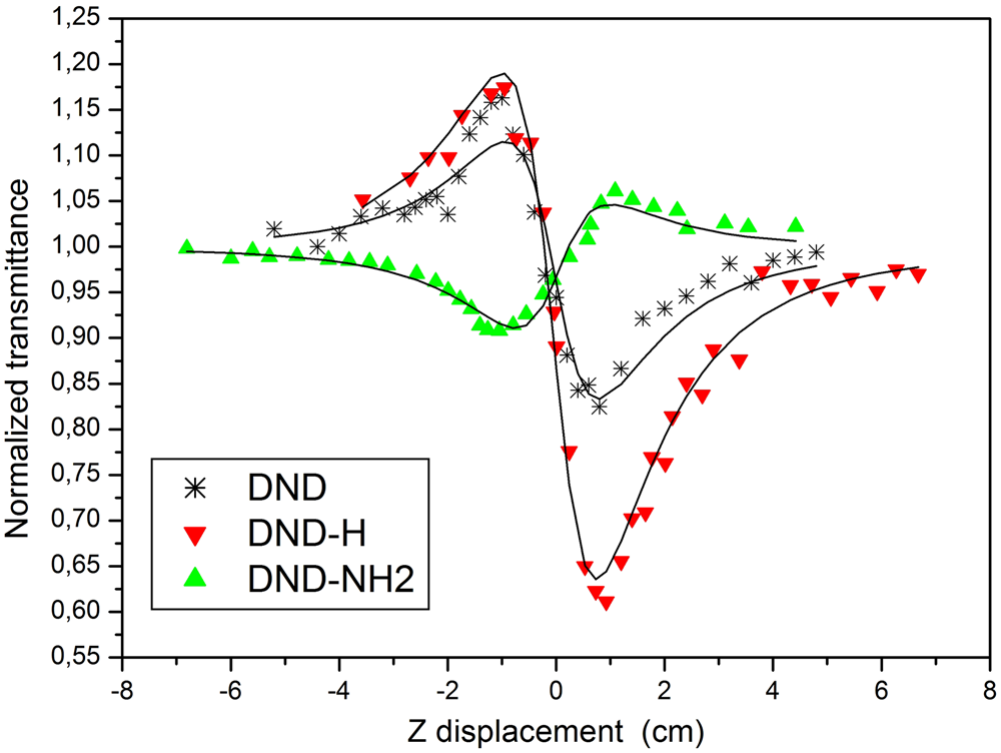
Close Z-scan signatures of the DND, DND-H and DND-NH2 at an incident laser fluence of F = 40 J/cm² (*I*_0_ = 10^10^ W/cm²) for the former suspension and F = 4 J/cm² (*I*_0_ = 10^9^ W/cm²) for both latter. The solid lines denote the theoretical fits of equation (8).

The Z-scan technique is a sensitive and reliable characterization method of the nonlinear optical properties of materials allowing the separation of nonlinear absorption and nonlinear refraction. This technique is thoroughly described in the study of Sheik-Bahae *et al*.^30^. In order to investigate the nonlinear refraction in our samples, the Z-scan in its closed aperture scheme was used (Fig. 4). The sensitivity of the measurement depends on the aperture factor S which has to be carefully defined. The appropriate value of S in our investigations is S = 2%; more details on the calculation of this parameter can be found in^15^. The close Z-scan experimental traces of the DND, DND-H and DND-NH2 are displayed on Fig. 6 together with the theoretical fits using the equations modeling the normalized transmittance when self-focusing or self-defocusing occur. The theoretical model based on the assessment of the real (Reχ^(3)^) and imaginary (Imχ^(3)^) parts of the third order nonlinear susceptibility has been first described by Mansour Sheik Bahae in 1990^30^ and used in other research groups (see e.g.^38,39^). The real and the imaginary parts of the third order nonlinear susceptibility are related to the nonlinear refraction,*n*_2_ and nonlinear absorption, β coefficients, respectively and are expressed as:

1 $${Re}{\chi }^{(3)}=2{n}_{0}^{2}{\varepsilon }_{0}c{n}_{2},$$

2 $${Im}{\chi }^{(3)}=\frac{{n}_{0}^{2}{\varepsilon }_{0}c\beta }{k},$$

where *n*_0_ is the linear refraction coefficient, *ε*_0_, the vacuum permittivity, *c* is the speed of light in vacuum and *k* denotes the wave number. The expression for the normalized transmittance, *T*(*z*), in our case can be written as:

3 $$T(z)=1+\frac{4a}{({a}^{2}+9)({a}^{2}+1)}{\rm{\Delta }}{\varphi }_{0}-\frac{2({a}^{2}+3)}{({a}^{2}+9)({a}^{2}+1)}{\rm{\Delta }}{\psi }_{0}.$$

In Equation (3), *a* = *z/z*_0_ with *z*, the linear displacement, whereas Δ*ϕ*_0_ and Δ*ψ*_0_ are the relevant parameters denoting the phase shift around the focal point as a result of the wave distortion due to nonlinear refraction and nonlinear absorption, respectively. Consequently, for both terms we can write:

4 $${\rm{\Delta }}{\varphi }_{0}=k{n}_{2}{I}_{0}{L}_{eff},$$

5 $${\rm{\Delta }}{\psi }_{0}=\beta {I}_{0}{L}_{eff}/2,$$

where *I*_0_ is the laser peak irradiance and *L*_*eff*_ the effective length of the sample defined through:

6 $${L}_{eff}=(1-{e}^{-\alpha L})/\alpha ,$$

where *L* and *α* represent the sample length and the linear absorption coefficient, respectively. Using quasi-similar experimental values (λ = 532 nm) *α* = 0.51 cm^−1^ (DND and DND-H) and *α* = 0.60 cm^−1^ (DND-NH2), a value *L*_*eff*_ = 0.097 cm^−1^ is deducted. A simplified form for relation (3) can be expressed after introducing the so-called coupling factor that follows^40^:

7 $$\rho =\frac{{Im}{\chi }^{(3)}}{{Re}{\chi }^{(3)}}=\frac{\beta }{2k{n}_{2}}=\frac{{\rm{\Delta }}{\psi }_{0}}{{\rm{\Delta }}{\varphi }_{0}}.$$

This brings for *T*(*z*):

8 $$T(z)=1+\frac{2(\,-\,\rho {a}^{2}+2a-3\rho )}{({a}^{2}+9)({a}^{2}+1)}{\rm{\Delta }}{\varphi }_{0}.$$

It is to be pointed out that the close aperture Z-scan results on Fig. 6 are generated at incident fluence levels of 4 J/cm² for the DND-H and DND-NH2 and 40 J/cm² for the DND related to the onset of nonlinear transmittance on Fig. 5.

A usual peak to valley shape is observed for the DND and the DND-H and expresses a self-defocusing effect consequently to a negative nonlinearity $(Re{\chi }^{(3)} < 0)$^15^, whereas, surprisingly, the DND-NH2 suspension exhibits a valley to peak profile. In this last case, the nonlinearity has changed sign and becomes positive $(Re{\chi }^{(3)} > 0)$ as explained by a self-focusing effect. Generally speaking, such a behavior is observed when nonlinear materials are submitted to ultrashort laser pulses^41–44^ and is currently attributed to Kerr effects nonlinearities. On the lecture of Fig. 6, it is obvious to emphasize the higher nonlinear refractive character of the DND-H system combined with the occurrence of significant nonlinear absorption revealed by the curve asymmetry. Furthermore, it is worth noting that the DND and DND-NH2 bring much weaker nonlinearities. By virtue of relation (8), the values of *n*_2_ were calculated to be −(6.0 ± 0.3) × 10^−15^ cm²/W for the DND, −(1.2 ± 0.1) × 10^−13^ cm²/W for the DND-H and +(2.9 ± 0.1) × 10^−14^ cm²/W for the DND-NH2. Concerning the DND-H system, it is reasonable to assume a two components contribution to the nonlinear refractive index as a consequence of thermal lensing effects and the electronic Kerr effect. According to the significantly lower value of *n*_2_ calculated in the case of the DND suspension, the hypothesis for thermal-induced refractive index changes seems to be correct. Indeed, the thermal lensing consists of a fast process of acoustic waves propagation and a slow steady state variation of the suspension density due to the cumulative laser-induced thermal heating of the absorbing area. It is interesting to notice that the present observations do not match those from a previous work^15^ which demonstrated the predominance of the Kerr nonlinearities but in a system composed of a another dispersant with different particle size. On the other hand, assuming that intra and intermolecular charges transfer occur most likely in the DND-NH2, the sign change of the nonlinear refractive index could be explained. However, such nonlinear phenomena take place on a sub-nanosecond timescale which cannot compare with the duration of the laser pulses of the present study. Accordingly, it might be possible that a tenfold occurrence of ultrashort nonlinearities where molecular reorientational Kerr effect cumulates with enhanced charges transfer could be responsible for the observed valley to peak shape. Indeed, the NH2 moiety could alternatively act as an electron donor (by conjugation) when the N atom, through its free electron pairs, participates in resonance, or as a withdrawing group if the N atom cannot participate in conjugation.

Following the same close Z-scan formalism, the nonlinear absorption coefficients β were calculated. The values are shown in Table 2 and compared to the β values calculated from the open Z-scan theory. Indeed, as the nonlinear refraction and absorption phenomena are simultaneously detected in the close-aperture scheme, the open-aperture optical arrangement enables to trace back the sole nonlinear absorption coefficient β. To the authors point of view it is mandatory to set up the couples of β values obtained from both methods and to compare them in order to discuss and validate the accuracy of the measurements. We used the equations governing the propagation of a Gaussian beam through the sample described in^30,45^. The expression for the open-aperture normalized transmittance is:

9 $$T(z)=\frac{\mathrm{ln}(1+q(z))}{q(z)},$$

where $q(z)=\beta I(z){L}_{eff}$, stands for the beam propagation parameter dependent on the β coefficient to be calculated and *I*(*z*) is the magnitude of the intensity of the Gaussian laser beam travelling in the +z direction. On the propagation axis for *r* = 0, it reduces to:

10 $$I(z)={I}_{0}\,\exp (\,-\,\gamma )/(1+\frac{{z}^{2}}{{z}_{0}^{2}}).$$

**Table 2:** Nonlinear absorption coefficients β calculated from the close Z-scan (left) and open Z-scan (right) methods.

Material	β data (cm/W)	
DND	(1.1 ± 0.3) × 10^−10^	(3.2 ± 0.5) × 10^−10^
DND-H	(4.2 ± 0.1) × 10^−9^	(4.5 ± 0.1) × 10^−9^
DND-NH2	(1.0 ± 0.0) × 10^−9^	(9.7 ± 0.0) × 10^−10a^

^a^The fitting errors are less than 0.1: Δ β = 0.03 (close Z-scan) and Δ β = 0.06 (open Z-scan).

**Figure 7:**
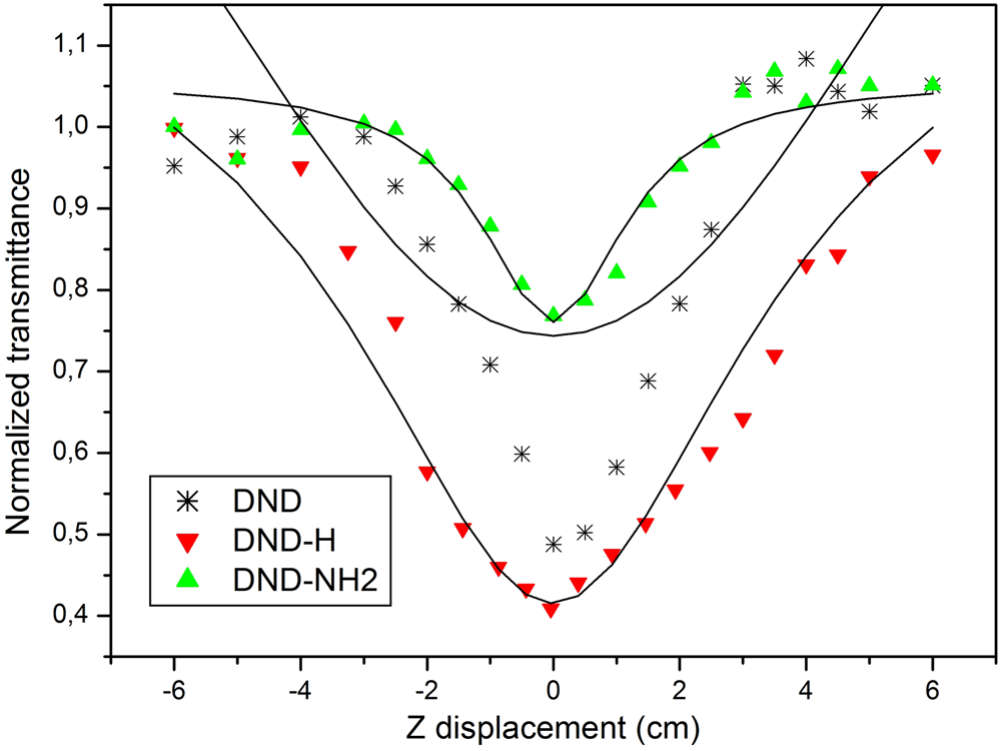
Normalized transmittances in the open Z-scan configuration for the DND suspension at an incident laser fluence of F = 40 J/cm² (*I*_0_ = 10^10^ W/cm²), and for the DND-H and DND-NH2 suspensions at F = 4 J/cm² (*I*_0_ = 10^9^ W/cm²). The solid lines denote the theoretical fits of equation (11).

In equation (10), we modified the conventional irradiance distribution *I*(*z*) by considering an additional parameter *γ* that expresses the extinction of the laser radiation in the propagating medium caused by scattering centers or defects. By substituting in relation (9), the expression for *T*(*z*) can be written again,

11 $$T(z)=\frac{{(1+{a}^{2})}^{2}}{\beta {I}_{0}{L}_{eff}{e}^{-\gamma }}\,\mathrm{ln}(1+\frac{\beta {I}_{0}{L}_{eff}{e}^{-\gamma }}{{(1+{a}^{2})}^{2}}),$$

where likewise in relation (3), *a* = *z*/*z*_0_. The results are displayed on Fig. 7 showing that the theoretical transmittances well match the experimental results with the exception of the DND suspension where a strong discrepancy can be observed. The calculated nonlinear absorption coefficients are summarized in Table 2.

**Figure 8:**
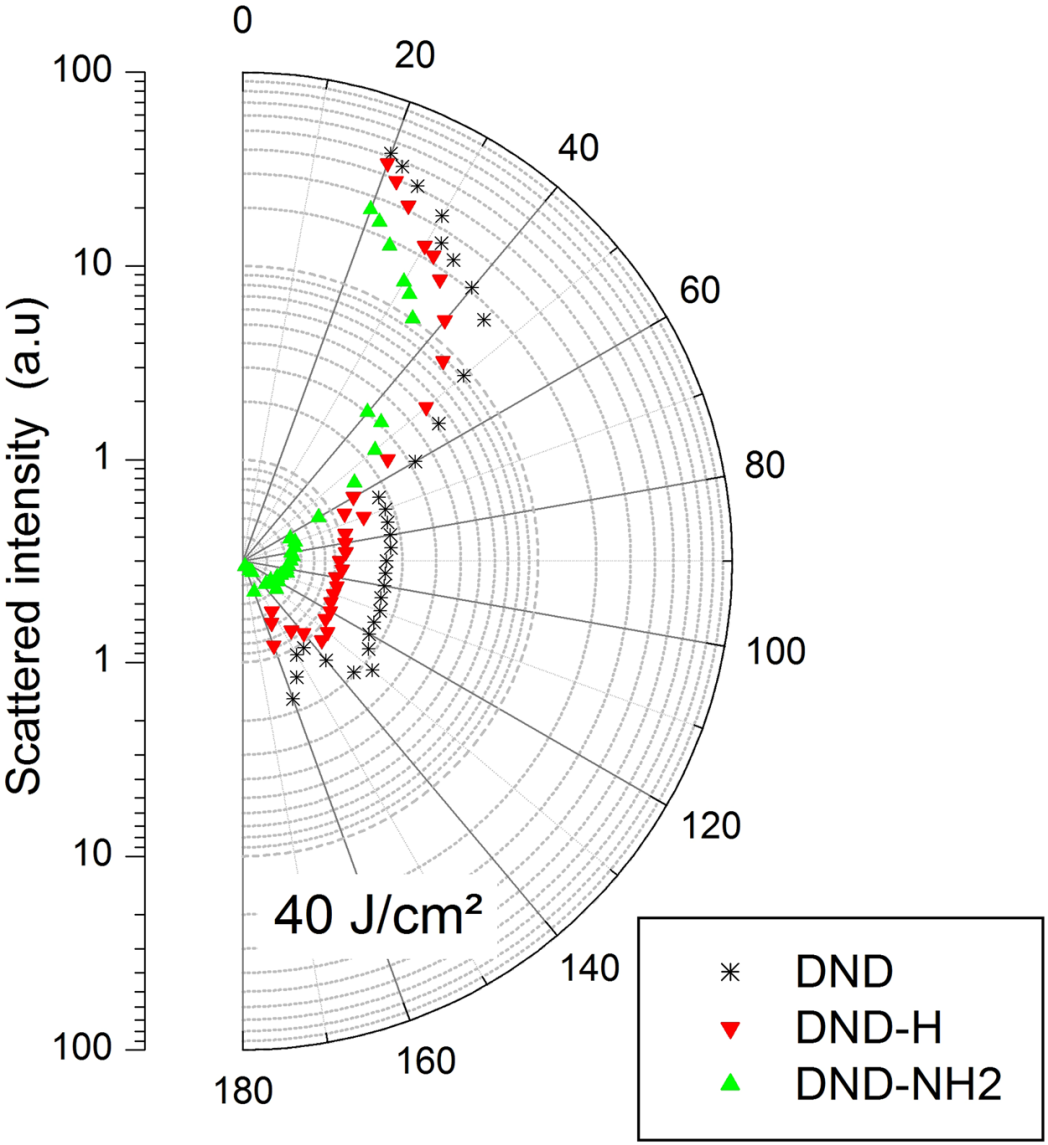
Semilog plot of the angular distribution of the scattered intensity for the DND, DND-H and DND-NH2 suspensions at F = 40 J/cm². The laser radiation is incident from the bottom to the top.

The β coefficients calculated using either method are in good agreement except for the DND as revealed in Table 2. While only DND has a poor fitting, the most important thing is, this bad fitting gives an unreliable β. Actually, the DND case is very interesting since none of the two theoretical models accurately fit the experimental results. Especially, what is clearly evidenced in Fig. 6 is the significant asymmetry in the peak-to-valley shape with respect to the zero transmittance line. Such an effect has already been reported^27,46^. It is most likely that the occurrence of a further process such as nonlinear scattering could explain the observed discordances. An additional scattering experiment campaign has been conducted in order to verify this hypothesis. For in the case of DND-H and DND-NH2, the most severe laser exposure parameter setup has been chosen that is F = 40 J/cm², ten times the onset of nonlinear transmittance. The results are displayed on Fig. 8.

These latter corroborate our postulate related to the strong nonlinear scattering character of the DND sample. Especially of interest in optical limiting, the polar sector [50–160 dg] from which the scattered radiation is rejected off the forward direction. Figure 8 confirms our predictions, showing that the strongest scattered signal is measured on the DND system in agreement with a previous study^15^. On the other hand, the signals measured on both other systems are much weaker, especially for the DND-NH2 compound, accrediting the nonlinear absorptive character of these species. Dealing with conventional nano-sized diamond crystals, a number of atoms near the surface bearing sp^2^ type bonding in association to bulky atoms with conventional sp^3^ orbitals have to be accounted for. As a consequence of the four-fold atoms coordination, a large energy bandgap of 5 eV can be expected^47^. On the other hand, when sp^2^ orbitals are considered, numerous energy states are created in the gap thus encouraging the optical absorption in the visible range. In view of the outstanding NEA surface of DND-H and DND-NH2, additional up- and downward valence and conduction band-bending and thus a diminution of the band gap can be conjectured^48^. In an attempt to verify this postulate that could explain the stronger nonlinear absorption of these compounds, we performed band gap measurements according to the absorption spectrum fitting method using the Tauc model well described in^49^. The results are stringent since band gap values of 3.4 ± 0.5 eV (DND-NH2) and 4.0 ± 0.5 eV (DND-H) were obtained, whereas the quasi-expected value was measured on the DND system (4.4 ± 0.5 eV). The conjunction of a lower band gap and the occurrence of additional energy states in the forbidden region are certainly responsible for the observed enhanced absorption at incident photon energy of 2.3 eV (i.e. 532 nm). Additional energy states may also arise from impurities and defects whereas band to band absorption can also be phonon-assisted in the manner of indirect band gap semiconductors. Further investigations are needed to clarify the absorption model of these NEA type DNDs.

**Figure 9:**
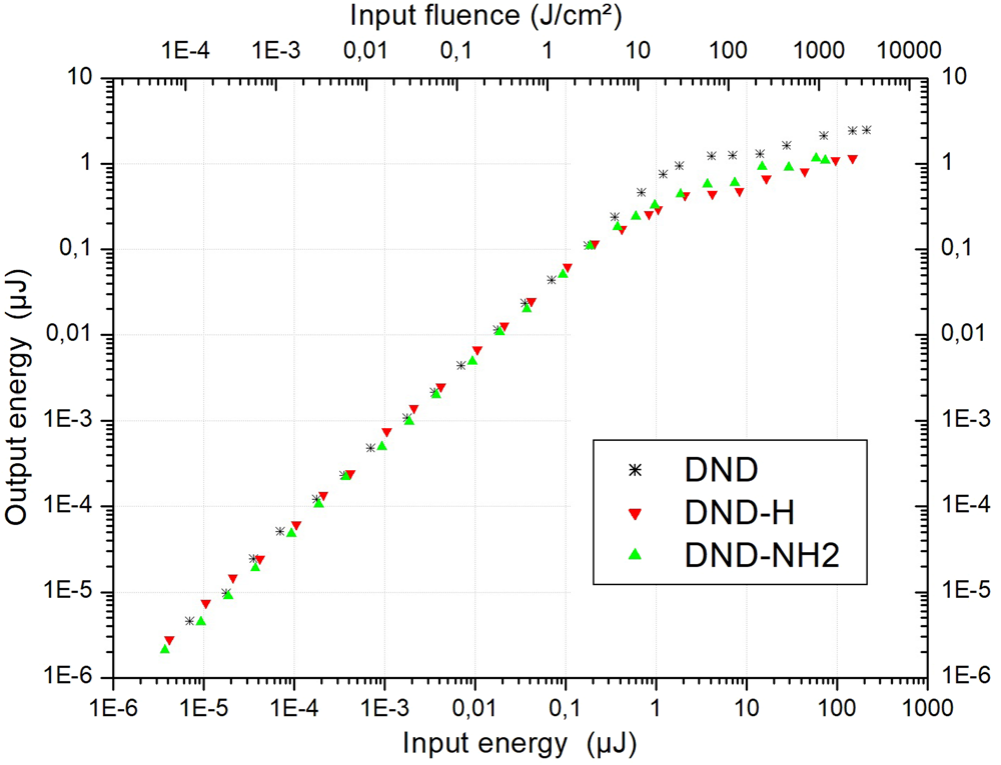
Output energy as a function of the input energy and input fluence in a log-log scale. DND, DND-H and DND-NH2 suspensions in water at λ = 532 nm are represented.

The optical limiting results can be discussed considering Figs 5 and 9, displaying the output energy as a function of the input energy for the 3 suspensions. The statement that the linear transmittance of an optical limiting filter has to exceed 40%^50^ is met for the 3 kind of suspensions (see Fig. 4, e.g.). The performance of a laser protection filter may be quantified through its global attenuation factor and is easily obtained by a direct lecture of the maximum transmittance excursion on Fig. 5. In this way, the maximum amplitude gives an attenuation of OD = 1.8 for the unfunctionalized DNDs, whereas we obtain attenuation values of OD = 1.9 and OD = 1.5 for the DND-H and DND-NH2 suspensions, respectively. Concerning our experimental findings, we notice that the largest nonlinear attenuation is related to the system exhibiting the lowest threshold value (i.e. the DND-H one). Above the threshold, the output energy variations strongly bend so that the transmitted laser radiations leaving the optical limiter could be fixed below a level of 2.5 µJ (DND) and approximately 1.0 µJ in the case of the DND-H and DND-NH2 suspensions. For the human eye, the transmitted laser radiation should be below the maximum permissible exposure (MPE), which is 0.33 μJ/cm² for nanosecond laser pulses at the wavelength of 532 nm^51^. This value is calculated using a laser repetition frequency of 20 Hz and assuming an exposure time of 0.25 s which is the duration of a blinking reflex. The MPE corresponds to a deposited laser energy on the retina of ca. 0.2 μJ for a fully dilated pupil (7 mm in dark vision). It is worth saying that in most of the optical limiting experiments this value cannot be reached, therefore it is reasonable to argue that the transmitted laser radiation should not exceed the ED50 value, which is the value for 50% probability of a retinal damage. The ED50 value is approximately ten times higher than the MPE^52^. For example, for 15 ns laser pulses at the wavelength of 532 nm an ED50 value of 3 μJ was found^51^. If we consider a value ED50 = 2 µJ, both of the DND-H and DND-NH2 suspensions are compliant with this eye protection level while the one for the unfunctionalized DND suspension lies slightly above. The last but not least point concerns the damage threshold of the optical limiter. Generally speaking, the higher the threshold, the best is the optical limiter. However, since an optical limiter device has to be located at the intermediate focal plane of an optical device, the occurrence of optical damage at high input energies is inexorable. The solution of the problem is the use of suspensions similar as those presented in this work, which have the advantage of self-healing.

## Conclusion

In this work we aimed to clarify the nonlinear optical mechanisms responsible for the optical limiting of suspensions composed of amino-terminated DNDs, hydrogen-terminated DNDs and compared them to unfunctionalized DNDs. To the best of the authors’ knowledge, the nonlinear optical response of NEA type DNDs has never been reported. The largest nonlinear attenuation was observed on the DND-H system, whereas the exceedingly low threshold values for optical limiting for the DND-H and the DND-NH2 systems were attributed to their NEA character, explicitly electron donor ability due to adequate surface dipoles. Z-scan experiments performed in a close aperture scheme have shown the higher nonlinear refractive character of the DND-H suspension combined with a significant nonlinear absorption, while the pure thermal origin of the nonlinear refractive index change could be readily conjectured in the case of the DNDs. On the other hand, an astonishing valley to peak profile was measured on DND-NH2 (*Reχ*^(3)^ > 0) which was attributed to the simultaneous presence of molecular reorientational Kerr effect and intra and intermolecular charges transfer due to the presence of the amino moiety. The specific nonlinear absorption for the DND-H and the DND-NH2 was explained in terms of a model based on the combination of a reduced band gap energy and additional states in the forbidden region. Two theoretical models describing the distribution of the transmittance *T*(*z*) through the probe could accurately fit the experimental data except for the case of the conventional DNDs. In agreement with^15^, it has been experimentally demonstrated that a significant nonlinear backscattering explains the observed discordance. The experimental findings reported in this study proof the inexhaustible aptitudes of nanodiamonds in generating singular nonlinear effects especially when surface chemistry is used. Regarding the optical limiting properties, the attenuation of the transmitted laser radiation is close to OD = 2.0 for the DNDs and the DND-H systems, whereas it lasts at a value of OD = 1.5 in the case of the DND-NH2 suspension. We also notice that the largest nonlinear attenuation is related to the system exhibiting the lowest threshold value (i.e. the DND-H system). Additionally, we saw that the transmitted laser radiation strongly bent and accordingly could be limited at a level below 1.0 µJ in the case of the DND-H and DND-NH2 suspensions. Although this latter value remains still above the MPE for the human eye, the compliance with the ED50 eye protection level is met with both of the aforementioned suspensions.
